# Modeling the Health Economic Burden of Hepatitis C Virus Infection in Turkey: Cost-Effectiveness of Targeted Screening

**DOI:** 10.5152/tjg.2023.22749

**Published:** 2023-10-01

**Authors:** Ayhan Hilmi Çekin, Rahmet Güner, Ahmet Çağkan İnkaya, Dilek Oğuz, Oktay Özdemir, Ömer Fehmi Tabak

**Affiliations:** 1 Department of Gastroenterology, Health Sciences University Antalya Training and Research Hospital, Antalya, Turkey; 2 Department of Infectious Diseases and Clinical Microbiology, Ministry of Health Ankara City Hospital, Ankara, Turkey; 3 Department of Infectious Diseases and Clinical Microbiology, Hacettepe University Faculty of Medicine, Ankara, Turkey; 4 Department of Gastroenterology, Güven Hospital, Ankara, Turkey; 5 Department of Medical Education, İstanbul Health and Technology University, İstanbul, Turkey; 6 Department of Infectious Diseases and Clinical Microbiology, İstanbul University Cerrahpaşa Faculty of Medicine, İstanbul, Turkey

**Keywords:** Hepatitis C, model of care, health policy, economic burden, disease burden, screening

## Abstract

**Background/Aims:**

In 2016, World Health Organization introduced global goals to eliminate hepatitis C virus by 2030. The aim of this study is to analyze the epidemiologic and economic burden of hepatitis C virus in Turkey and compare current practice (regular care) with a hypothetical active screening and treatment approach (active scenario).

**Materials and Methods:**

A Markov model was used to analyze and compare regular care with a scenario developed by experts including the screening and treatment of all acute and chronic hepatitis C virus infections between 2020 and 2050. General and targeted populations were focused. The model reflected the natural history of the disease, and the inputs were based on a literature review and expert opinions. Costs were provided by previous studies and national regulations.

**Results:**

The active scenario resulted in higher spending for all groups compared with regular care in the first year. Cumulative costs were equalized in the 8th, 12th, 13th, and 16th year and followed by cost-savings of 49.7 million, 1.1 billion, 288.6 million, and 883.4 million Turkish liras in 20 years for prisoners, refugees, people who inject drugs (PWID), and all population, respectively. In all groups, the mortality was found to be lower with the active scenario. In total, 62.8% and 50.6% of expected deaths with regular care in 5 and 20 years, respectively, were prevented with the active scenario.

**Conclusions:**

An active screening and treatment approach for hepatitis C virus infection could be cost-effective for PWID, prisoners, and refugees. Almost two-thirds of deaths in regular care could be prevented in 5 years’ time with this approach.

**Main Points**

- Underdiagnosis of hepatitis C virus (HCV) infection is a concern. Particular attention needs to be focused on population groups with a higher prevalence of HCV, such as people who inject drugs, prisoners, refugees, and people with risky sexual behavior.
- We analyzed the epidemiologic and economic burden of HCV in Turkey and compared current practice with a hypothetical active scenario which included the screening and treatment of all acute and chronic HCV-infected patients.
- Active scenario was found to be cost-effective for people who inject drugs, prisoners, and refugees. Almost two-thirds of HCV-related deaths could be prevented in 5 years’ time with this approach.

## Introduction

Globally, an estimated 71 million people have chronic hepatitis C virus (HCV) infection, and the risk for cirrhosis in people with chronic HCV infection is 15%-30% within 20 years. The World Health Organization (WHO) also estimated that in 2016, approximately 399 000 people died from HCV, mostly owing to complications of cirrhosis and hepatocellular carcinoma.^1^

Substantial progress in the treatment of HCV has been made since the introduction of direct-acting antivirals (DAAs) in 2013, resulting in improved efficacy and tolerance and a shorter duration of treatment compared with previous treatments.^2^ However, challenges in the treatment of HCV remain. Underdiagnosis of HCV infection is still a concern: it is estimated that only 20% of people with HCV worldwide have been diagnosed.^2^ Additionally, particular attention needs to be focused on those population groups with a higher prevalence of HCV, such as people who inject drugs (PWID), prisoners, refugees, and men who have sex with men (MSM). Preventing transfusion-related HCV transmission has been identified as a priority, and blood transfusion safety has improved since 2000.^2,3^ In 2016, with the success of DAAs, WHO introduced global goals for the care and management of HCV: a 90% reduction in new cases of chronic HCV, a 65% reduction in HCV-related deaths, and treatment of 80% of eligible people with chronic HCV infection by 2030.^4^ Unfortunately, current levels of testing and treatment are generally insufficient to achieve these goals in most settings. Globally, elimination scenarios and economical model strategies have been studied to help achieve these goals.^3,5-7^

In this study, our aim was to analyze the epidemiologic and economic burden of HCV in Turkey and compare current practice with a hypothetical active screening and treatment approach.

## Materials and Methods

Analyses of the disease burden of HCV in Turkey were based on a Markov model built in Microsoft Excel^®^.^8^ Our primary objective was to analyze the cost-effectiveness of an active screening and treatment approach (referred as the active scenario) in targeted populations, which comprised blood transfusion recipients before 2000, PWID, MSM, prisoners, and refugees. Secondary objectives were to investigate the cost-effectiveness of the active scenario in the general population (defined as the non–high-risk population that remains after exclusion of the targeted populations) and the reduction of mortality in the general as well as targeted populations, with the active scenario compared with current practices (referred to as regular care). A panel meeting was held in Ankara in December 2019 and an online meeting in December 2020 consisting of 5 physicians from infectious disease and gastroenterology specialties and one model/analysis specialist to reach a consensus on all inputs of the model.

Regular care represented the treatments currently used to manage HCV infection. The disease model reflected the natural history of acute and chronic HCV infection.^9^ The active scenario, which was created by panel participants, comprised the following criteria: (i) anti-HCV antibody testing for all patients with acute HCV infection, (ii) HCV-RNA testing for all those with positive anti-HCV antibody tests, (iii) treatment for all patients with positive HCV-RNA tests, and (iv) diagnosis and (v) treatment of all chronic HCV infections. Data for the total adult population were obtained from the United Nations Department of Economic and Social Affairs,^10^ and the ratio of blood transfusion recipients was estimated by panel participants. The PWID prevalence data were gathered from the 2019 Turkish Drug Report^11^ and data regarding the number of prisoners were from Turkish Statistical Institute reports.^12^ Population of MSM was calculated based on Marcus et al,^13^ and the number of refugees was obtained from the United Nations Refugee Agency.^14^ Mortality calculations were based on the number of individuals with chronic HCV and estimates for progression rates to cirrhosis, decompensated cirrhosis, hepatocellular carcinoma, and liver transplantation. Estimations used as model input for current acute and chronic HCV infection are presented in Tables 1 and 2 with references.^15-59^

Healthcare service costs were calculated using the Official Health Notification (December 2020) of the Social Security Institution of Turkey.^60^ These services included diagnostic tests, such as anti-HCV antibody, HCV-RNA, and HCV genotyping tests. Medication costs were estimated by panel participants, and costs of other health states (chronic hepatitis, cirrhosis, decompensated cirrhosis, hepatocellular carcinoma, and liver transplantation) were based on previous studies^61,62^ and used with currency and inflation adjustments. Costs of diagnostic tests were calculated as 8.62 Turkish liras (TL) for an anti-HCV antibody test, 111.87 TL for an HCV-RNA test, and 109.59 TL for an HCV genotyping test. Annual medication cost was estimated at 15,000 TL. Other annual medical cost estimates were 1502 TL for chronic hepatitis, 1546 TL for cirrhosis, 12,512 TL for decompensated cirrhosis, 39,749 TL for hepatocellular carcinoma, and 169,350 TL for liver transplantation.

The cost of tests for screening purposes, the cost of medication for patients receiving treatment, and other treatment costs for patients who were non-responders or non-compliant with the treatment or who did not receive any treatment were collected, and a total cost calculation was made. Incremental cost-effectiveness ratios were calculated in terms of death averted. The model was projected between 2020 and 2050.

## Results

The total adult population of Turkey in 2019 was 62 million. Individuals with HCV are shown in Table 3 along with their treatment status. It was calculated that there were 1029 people with acute and 592 970 with chronic HCV infection. Of these 1029 people with acute HCV infection, 23 (2.24%) were estimated to receive treatment, whereas 213 469 (36.0%) people with chronic infection were estimated to be under treatment.

**Figure 1.**
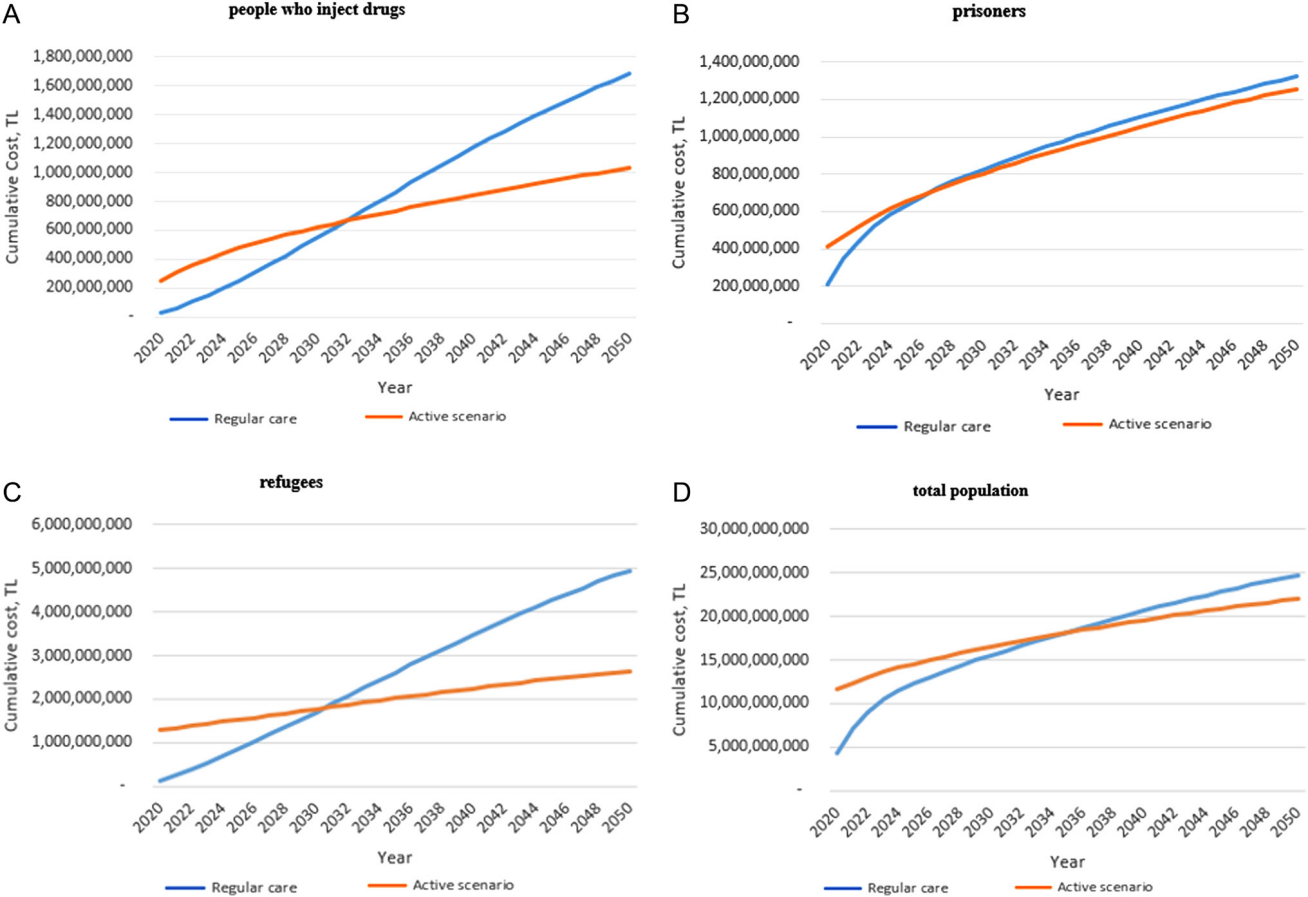
Cumulative total costs of HCV management with regular care and active scenario for (A) people who inject drugs, (B) prisoners, (C) refugees, and (D) total population. HCV, hepatitis C virus; TL, Turkish liras.

Calculated cumulative costs for the general population and targeted groups in the case of regular care and active scenario are given in Table 4 for 1, 5, and 20 years. The active scenario resulted in higher spending for the general population and all targeted groups compared with regular care in the first year. The highest cost saving was seen in the refugees group. For refugees, cumulative costs were equalized in the twelfth year, followed by cost savings of 1.1 billion TL by 2040. For the PWID group, cumulative costs were equalized in the thirteenth year, and a cost saving of 288.6 million TL was achieved by 2040. Similarly, cumulative costs were equalized in the eighth year and a cost saving of 49.7 million TL was achieved for prisoners with HCV by 2040. No significant cost saving was observed in MSM, blood transfusion, and general population groups (Table 4). When all people with HCV were considered, the costs were equalized from the 16th year onward, and 883.4 million TL were saved at 20 years. Cost savings over the years in PWID, prisoners, refugees, and the total population are given in Figure 1.

Estimated cumulative mortality for regular care and the active scenario are given in Table 5. In all groups, the mortality was found to be lower with the active scenario. The highest reduction (73.4%) in mortality rate in 5 years was observed in refugees, followed by PWID (68.1%), while the lowest reduction was in the MSM group (20.7%). In total, 62.8% and 50.6% of expected deaths with regular care in 5 and 20 years, respectively, were prevented with the active scenario.

## Discussion

In our study, we found that an active screening and treatment program for HCV infection in Turkey would be cost-effective for the total population as well as the higher-risk groups of PWID, prisoners, and refugees in 8 to 16 years. However, no significant cost saving was observed in the MSM group. A reduction in mortality rates in patients with HCV was anticipated: half of all deaths in these patients over 20 years could be prevented with the active scenario.

One of the most at-risk groups for HCV infection is PWID. This group had the highest incidence rate of all groups in this study and the lowest rate of treatment access after refugees. It has been estimated that there are 15.6 million PWID globally and that 52.3% are HCV-antibody positive.^63^ Controlling HCV infection in PWID is a focal point for WHO in combating HCV.^4^ Although sterile syringe/needle programs are an important step for harm reduction, active screening and treatment are also crucial for the prevention of HCV in this group. In Iceland, a program was launched in January 2016 aiming to provide treatment to all patients infected with HCV.^64^ The program, which was primarily focused on PWID, includes screening and DAA treatment as well as harm reduction and education. With these efforts, Iceland is anticipated to achieve HCV elimination goals well before the WHO goal of 2030. In our study, it was cost-effective after 13 years to launch an active scenario approach for PWID. Despite spending being almost 9 times higher than with regular care in the first year, the cumulative cost was favorable with the active scenario after 12 years. However, cultural and social differences among countries should be taken into consideration, and HCV screening and treatment programs should be tailored for PWID groups. The PWID status is also related to incarceration history, meaning that these 2 targeted groups, PWID and prisoners, could overlap. Degenhardt et al^63^ showed that 57.9% of PWID had a history of incarceration. Stone et al^65^ stated that recent and past incarcerations were associated with a 62% and 21% increase in HCV acquisition risk, respectively. Therefore, active screening and treatment efforts for one group could be of benefit to the other.

Prisoners have an increased risk of HCV transmission because of the continued use of drugs and shared syringes, getting new tattoos, and other incidents that involve contact with blood.^41^ There are several studies in the literature about the economic burden and the level of cost-effectiveness of scaling-up HCV screening and treatment among prisoners. In a study by He et al,^66^ it is shown that risk- or time-based screening scenarios could prevent 5500 to 12 700 new HCV infections and 4200 to 11 700 deaths related to liver diseases compared with no screening. Prisons, however, would require an additional 12.4% of their current health budget to implement such interventions.^66^ In addition to screening programs, treatment with DAAs would be cost-effective at various levels.^67-69^ In our study, the active scenario was beneficial at cumulative cost levels in the medium term (starting from the eighth year) in prisoners. However, because prisoners are a more isolated and controllable group, it may be preferable to prioritize other targeted groups for active scenario from an economic point of view.

Turkey hosts over 3.6 million refugees, the largest number for any country.^14^ Refugees could be a difficult group to engage because of issues such as limited available HCV data, their reluctance to volunteer for testing, and the difficulties inherent in accessing treatment in a foreign country.^70^ Data regarding the cost-effectiveness of HCV treatment in refugees varied in the literature, depending on the treatment.^71^ In our study, the cost of care in the active scenario was almost 10 times the expenditure of regular care in the first year, but, beginning with the twelfth year, the costs equalized, resulting in a 1.1 billion TL benefit in 20 years. Special issues for refugees are also within the focus of national health authorities, and further studies are needed.

In previous studies, hepatitis C screening in the MSM group has been shown to be cost-effective.^72,73^ Additionally, treatment with DAAs was found to be also effective to reduce HCV infection among human immunodeficiency virus (HIV)-positive MSM.^74,75^ Despite being one of the targeted groups, the active scenario was not cost-effective in 5 and 20 years of expenditures for MSM in our study. However, in a meta-analysis, HCV was found to be highly associated with HIV and drug injection in the MSM group.^76^ Therefore, overlap between targeted groups should be considered for economic evaluation.

The most prominent reduction in mortality at 5 and 20 years was found among refugees, followed by PWID. Almost two-thirds of deaths for patients in regular care could have been prevented in 5 years’ time with the active scenario, reaching the WHO goal of a 65% reduction in mortality by 2030.^4^

There are a few limitations. Panel discussions were used to provide model input estimates for which real-world data were not available. Economic losses due to loss of workforce, indirect expenses, new cases of HCV infection in the community caused by the presence of infected individuals, and a quality-of-life analysis of chronic HCV infection periods were not included.

In conclusion, an active screening and treatment approach for HCV infection could be cost-effective in the medium term for PWID, prisoners, and refugees. For the total HCV-infected population, it could mean an 883.4 million TL saving in 20 years. Because prisoners are more isolated, it might be preferable to focus on PWID and refugees first to ease the short-term economic burden of the active program. Combating HCV in the general and targeted populations requires the attention of medical professionals as well as socioeconomic experts and policymakers.

## Figures and Tables

**Table 1.** Population Estimates Used as Model Input

Population Groups	Acute HCV infection, %					Chronic HCV infection, %				
	Incidence	Anti-HCV Antibody Testing	HCV-RNA Testing of Anti-HCV Antibody Positive Patients	HCV-RNA Positivity After 3 Months	Follow-Up and Treatment of Patients with Continued HCV-RNA Positivity	Prevalence	Awareness of HCV Infection	Follow-Up and Treatment of Aware Patients	Treatment Compliance	Cure
General population	0.0018^15,16^	10^*^	53.8^17-22^	65.6^17,20-23^	63.5^17,20,22^	1.01^23^^-^^28^	40^*^	90^*^	87^*^	98^*^
Targeted population										
Blood transfusion recipients	0.0018^*^	10^*^	53.8^*^	65.6^*^	63.5^*^	1.01^*^	40^*^	90^*^	87^*^	98^*^
PWID	24.0^15,29-31^	10^*^	90^*^	65.6^*^	10^*^	49.2^10,32-35^	10^*^	20^*^	70.9^36^	98^*^
MSM	0.148^37^	10^*^	90^*^	65.6^*^	80^*^	1.06^38^	60^*^	90^*^	75^*^	98^*^
Prisoners	1.55^30,31^	10^*^	80^*^	73.9^39^	79.8^39^	6.6^39,40-42^	60^36,39^	90^*^	75.8^36,39^	98^*^
Refugees	0.0018^*^	10^*^	70^*^	65.6^*^	50^*^	4.1^43-46^	3^*^	3^*^	75^*^	98^*^

HCV, hepatitis C virus; MSM, men who have sex with men; PWID, people who inject drugs.

^*^Panel estimation.

**Table 2.** Estimates for Natural History of HCV Infection Across Disease States

	Disease state, %					
	Chronic Hepatitis	Cirrhosis	Decompensated Cirrhosis	HCC	Liver Transplantation	Mortality
Chronic hepatitis^*^	95.58	4.17	0.00	0.25	0.00	0.00
Cirrhosis	-	91.35	4.32^47-51^	1.48^47,48,50,51,53^	0.00*	2.85^47,48^
Decompensated cirrhosis	-	-	71.76	1.84^50-53^	2.44^49-51,53^	23.96^47-52^
HCC	-	-	-	45.44	0.00^50,51,53^	54.56^47,49-51,53,54^
Liver transplantation	-	-	-	-	-	14.43^55-59^

HCC, hepatocellular carcinoma; HCV, hepatitis C virus.

^*^Panel estimation.

**Table 3.** Total and HCV populations in Turkey according to the treatment status

Population groups	Total	HCV	Patients receiving treatment	Non-responder or non-compliant	Untreated
Targeted population					
Refugees	2 006 606	82 673	75	20	82 586
Prisoners	338 125	27 663	12 355	3172	13 939
PWID	24 920	18 255	281	86	15 915
MSM	292 212	3534	1696	449	1690
Blood transfusion recipients	600 534	6067	2181	321	3883
General population	58 797 622	593 999	213 492	31 469	380 153
Total	62 060 019	732 191	230 079	35 517	498 166

HCV, hepatitis C virus; MSM, men who have sex with men; PWID, people who inject drugs.

All numbers were given as sum of people with acute and chronic HCV infection.

**Table 4.** Cumulative Costs of Regular Care and Active Scenario in the General and Targeted Groups

	Cumulative Costs, TL								
Population Groups	1 Year			5 Years			20 years		
	Regular Care	Active Scenario	Difference	Regular Care	Active Scenario	Difference	Regular Care	Active Scenario	Difference
Targeted population									
Refugees	125 185 714	1 290 119 618	1 164 933 904	696 865 481	1 482 522 814	785 657 333	3 307 919 255	2 204 093 228	−1 103 826 027
Prisoners	211 113 578	408 549 353	197 435 776	581 715 631	612 028 624	30 312 993	1 080 566 875	1 030 831 172	−49 735 703
PWID	28 352 667	251 758 813	223 406 146	198 710 672	440 740 047	242 029 375	1 110 057 408	821 419 897	−288 637 510
MSM	28 654 244	54 738 983	26 084 740	68 623 547	77 902 189	9 278 642	119 097 424	125 234 789	6 137 365
Blood transfusion recipients	39 021 705	97 469 566	58 447 861	100 654 216	116 101 263	15 447 047	147 247 451	152 835 089	5 587 638
General population	3 820 571 266	9 543 135 721	5 722 564 455	9 854 941 244	11 367 344 289	1 512 403 045	14 416 832 580	14 963 911 971	547 079 391
Total	4 252 899 173	11 645 772 054	7 392 872 882	11 501 510 791	14 096 639 227	2 595 128 436	20 181 720 992	19 298 326 146	−883 394 846

MSM, men who have sex with men; PWID, people who inject drugs; TL, Turkish liras.

**Table 5.** Mortality with Regular Care and Active Scenario Approach of the Groups at 5 and 20 Years

Population Groups	Mortality					
	5 Years			20 Years		
	Regular Care, n	Active Scenario, n	Difference, n (%)	Regular Care, n	Active scenario, n	Difference, n (%)
Targeted population						
Refugees	1977	525	1452 (73.4)	25 169	6704	18 464 (73.4)
Prisoners	232	179	53 (22.8)	3255	2875	380 (11.7)
PWID	439	140	299 (68.1)	7409	2488	4921 (66.4)
MSM	29	23	6 (20.7)	373	336	37 (9.9)
Blood transfusion recipients	54	21	33 (61.1)	442	274	168 (38.0)
General population	5307	2099	3208 (60.5)	43 294	26 843	16 451 (38.0)
Total	8038	2987	5050 (62.8)	79 942	39 521	40 422 (50.6)

MSM, men who have sex with men; PWID, people who inject drugs.
